# Clinical Studies Applying Cytokine-Induced Killer Cells for the Treatment of Renal Cell Carcinoma

**DOI:** 10.3390/cancers12092471

**Published:** 2020-09-01

**Authors:** Ying Zhang, Jörg Ellinger, Manuel Ritter, Ingo G. H. Schmidt-Wolf

**Affiliations:** 1 Department of Integrated Oncology, CIO Bonn, University Hospital Bonn, Venusberg-Campus 1, D 53127 Bonn, Germany; Ying.Zhang@ukbonn.de; 2 Department of Urology, University Hospital Bonn, Venusberg-Campus 1, D 53127 Bonn, Germany; joerg.ellinger@ukbonn.de (J.E.); mritter@ukbonn.de (M.R.)

**Keywords:** cytokine-induced killer cells, clinical study, renal cell carcinoma, immunotherapy, preclinical research

## Abstract

**Simple Summary:**

Cytokine-induced killer (CIK) cells are a heterogeneous population of polyclonal T effector cells expanded ex vivo. Here, we updated our last review published in 2012 and provided a synopsis of current 15 clinical studies, including 382 patients with renal cell carcinoma (RCC) enrolled in CIK cell immunotherapy. CIK cells exhibited promising synergistic anti-tumor effects when combined with conventional therapies and showed mild adverse effects in patients with RCC. Preclinical researches also identified potential molecular targets that augmented CIK cell cytotoxicity against renal carcinoma cells. In future, large randomized clinical trials should be organized to further evaluate the clinical efficacy and optimize the treatment modality of CIK cells in RCC.

**Abstract:**

There is growing interest in cytokine-induced killer (CIK) cells on the integrated therapy of patients with RCC, especially those in the late stage or refractory to conventional chemotherapy and radiotherapy. In this review, a total of 15 clinical studies including 681 patients enrolled in CIK cell immunotherapy were outlined. Three-hundred-and-eighty-two patients with RCC were treated with CIK cells alone or in combination with DC vaccination, targeted agents sunitinib or sorafenib, and the PD-1 inhibitor pembrolizumab. Significantly improved 3-year overall survival rate was reported in four trials, whereas remarkably longer median progression-free survival was observed in three studies. Adverse reactions were mild and usually controllable fever and fatigue. Besides, preclinical research progresses were reviewed to increase our understanding about the underlying mechanisms of CIK cell cytotoxicity and identify potential targets to enhance their anti-tumor activity. These studies suggest that CIK cell-based immunotherapy has potential clinical benefits with a good safety profile and could become a promising approach in the combined therapies of RCC patients. However, further large-scale studies are required to evaluate the clinical efficacy of CIK cells and more efforts should be performed to identify the optimal CIK cell-based therapeutic regimen for RCC patients.

## 1. Introduction

Renal cell carcinoma (RCC), among the 10 most frequently diagnosed cancers, acts as one of the most lethal urological malignancies worldwide [1]. The highest age-standardized incidence rate is in North America (11.7 per 100,000), with Western Europe ranking the second (9.8 per 100,000) [1]. Lots of patients with renal masses remain asymptomatic until the late stages and over 60% of RCCs are detected incidentally with abdominal imaging performed for other reasons [2]. Although most detected lesions are small tumors at the time of diagnosis, locally advanced disease continues to be diagnosed in a notable proportion of patients, with distant metastases present in up to 17% of patients [3].

Surgery is the only curative treatment for localized RCC, but metastatic RCC (mRCC) is usually refractory to conventional therapies [4]. For most patients with mRCC, systemic therapy, including targeted therapy and immunotherapy, is necessarily required [2]. There are several targeted drugs that have been approved for the treatment of mRCC. They mainly consist of inhibitors targeting vascular endothelial growth factor (VEGF) and its receptors (bevacizumab, sunitinib, sorafenib, pazopanib, axitinib, tivozanib and cabozantinib) and mammalian target of rapamycin (mTOR) (everolimus and temsirolimus) [5,6]. Until targeted therapies were introduced in 2006, the first-line treatment strategy for mRCC was immunotherapies based on interleukin-2 (IL-2) combined with interferon-α (IFN-α) [7]. In 2018, the CheckMate 214 study reported superiority of the programmed cell death-1(PD-1) inhibitor nivolumab and cytotoxic T-lymphocyte-associated antigen 4 (CTLA-4) inhibitor ipilimumab over sunitinib in intermediate- and poor-risk patients. A significantly higher overall survival (OS) and objective response rates were achieved with nivolumab plus ipilimumab than with sunitinib [8]. These findings resulted in an updated recommendation of the first-line management for mRCC patients.

**Figure 1:**
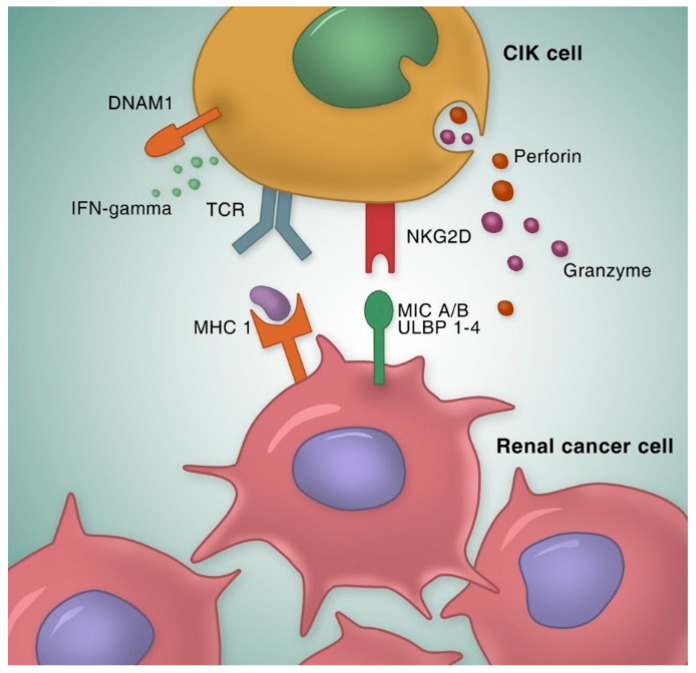
Mechanisms of CIK cell anti-tumor activity. The majority of CIK cell cytotoxicity is exerted through the interaction between NKG2D and its ligand MIC A/B and ULPB 1–4, resulting in granule exocytosis and secretion of cytokines like IFN-γ. NK-like structures, such as DNAM-1, is also involved in the recognition and killing of tumor targets. Besides, CIK cells can eradicate tumors in an MHC-restricted manner by TCR engagement, which is the so-called “dual-functional capability”. CIK cell, cytokine induced killer cells; NKG2D, natural killer group 2 member D; MIC A/B, MHC class I polypeptide-related sequence A/B; ULPB 1–4, UL16 binding protein 1–4. DNAM-1, DNAX accessory molecule-1; TCR, T-cell receptor; MHC 1, major histocompatibility complex 1; IFN-γ, interferon-γ.

In the past decades, there has been growing interest in adoptive cell-based immunotherapy as a therapeutic option in patients with RCC [9,10,11]. Cytokine-induced killer (CIK) cells, one of the main adoptive cell-based strategies, are a heterogeneous cell population comprising the main effector subset of double positive CD3 and CD56 cells. Schmidt-Wolf et al. first reported the generation of CIK cells ex vivo from peripheral blood mononuclear cells (PBMCs) in 1991, by adding IFN-γ on day 0 and monoclonal antibody against CD3 (anti-CD3 mAb), human recombinant IL-2 and IL-1 on the next day. An increased cytotoxicity of CIK cells against lymphoma was observed in a SCID mouse/human lymphoma model [12]. CIK cells exert a potent major histocompatibility complex (MHC)-unrestricted cytotoxicity against both hematological and solid malignancies with CD3+CD56+ cells as the main effectors. The natural killer group 2 member D (NKG2D) receptor appears to play the most important role in tumor recognition by CIK cells (Figure 1) [13]. NKG2D, a member of the c-type lectin-activating receptor family, recognizes the MHC-class I-like molecules, MICA and MICB and members of the ULBP family (ULPB1-4) that are restrictedly expressed on malignant tissues and mediates subsequently granzyme release [14]. It is also indicated that NK-like structures, including DNAX accessory molecule-1 (DNAM-1) and NKp30 are expressed on CIK cells and involved in the recognition and killing of tumor targets [15]. Furthermore, CIK cells showed reduced allo-reactive potential and minimal graft-versus-host disease (GVHD) activity in several in vitro and in vivo models, compared with conventional T cells [16,17]. This advantage makes CIK cells an appealing and promising alternative to classic donor lymphocyte infusion (DLI) after hematopoietic stem cell trans-plantation (HSCT).

**Figure 2:**
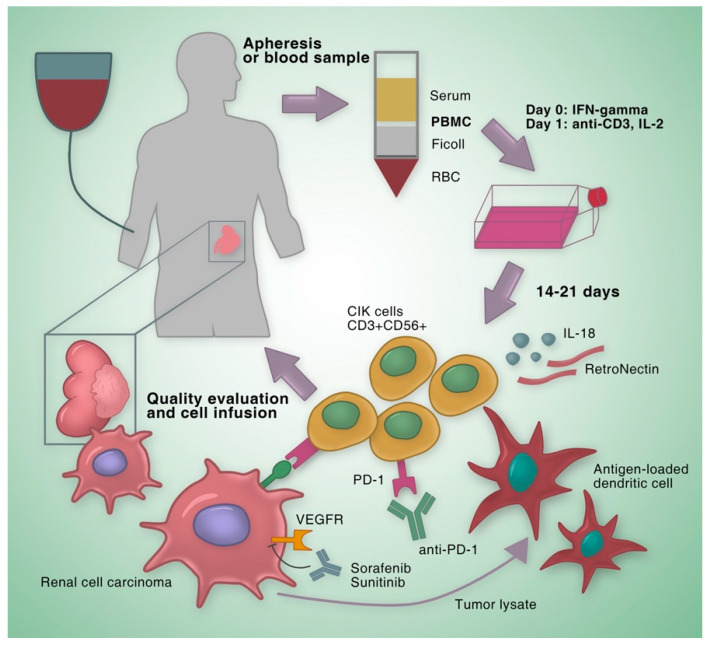
Strategies for improving clinical curative effects of CIK cells. PBMCs are isolated by density gradient centrifugation and expended with IFN-γ, anti-CD3 and IL-2. After 14–21 days, CIK cells are infused into the patient. The anti-tumor activity of CIK cells can be enhanced by adding cytokines like IL-18 or RetroNectin during expansion. DCs have high ability to present tumor antigens and compensate the lack of tumor antigen specificity of CIK cells. Targeted agents such as sunitinib and sorafenib or immune checkpoint inhibitor anti-PD-1 antibodies also have a synergistic effect with CIK cells. CIK cell, cytokine-induced killer cell; PBMC, peripheral blood mononuclear cell; IFN-γ, interferon-γ; IL-2, interleukin-2; IL-18, interleukin-18; VEGFR, vascular endothelial growth factor receptor; PD-1, programmed cell death-1; DC, dendritic cell; RBC, red blood cell.

In our last review published in 2012, we summarized eight clinical studies utilizing CIK cells for the treatment of RCC [18]. These studies showed that the combination of CIK cell therapy and standard therapy was superior to standard therapy alone. Since 2012, more efforts have been put to optimize the anti-tumor potency and treatment regimen of CIK cell-based therapies. In the following sections, we will provide an update on our former review by outlining new clinical results of RCC (Figure 2) and presenting progress of adoptive immunotherapy strategies based on CIK cells.

## 2. Clinical Studies on CIK Cells for the Treatment of RCC

A total of 15 clinical studies were reviewed here. Six-hundred-and-eight-one patients representing 55.4% of the total with distinct tumor entities were enrolled in CIK cell immunotherapy. Three-hundred-and-eighty-two patients with RCC were treated with CIK cells alone or in combination with DC vaccination, targeted agents sunitinib or sorafenib, and the PD-1 inhibitor pembrolizumab.

### 2.1. Clinical Studies on Autologous CIK cells

Twenty patients with unilateral, locally advanced (TNM stage I or II) RCC who had undergone radical nephrectomy were recruited into a study [19]. The patients were randomly divided into two groups, a CIK cell treatment group and a control group. In the CIK cell group, patients received 1–5 × 10^9^ autologous CIK cells intravenously per infusion and 2–10 × 10^9^ CIK cells in total for two consecutive days. Patients were also injected with 20 mg/day thymopentin intramuscularly before CIK cell transfusion and 1 mU rhIL-2 subcutaneously for 10 days after transfusion. 

Response Evaluation Criteria in Solid Tumors (RECIST) was used to assess the clinical response every 2 months. In the CIK cell treatment group, a complete response (CR) was achieved in six patients, a partial response (PR) was observed in two patients and stable disease (SD) was remained in two patients. Meanwhile, in the control group, a CR was achieved in five patients, SD remained in three patients, and progressive disease (PD) was observed in two patients. The median progression-free survival (PFS) in the CIK cell group was 32.2 months, which was significantly longer than that of 21.6 months in the control group. However, no statistically significant differences in OS were observed between two groups. All side effects were transient or controllable, including mild arthralgia, laryngeal edema, fatigue, and low-grade fever during lymphocyte infusion or during the early stages of rhIL-2 treatment. Grade 3 or greater adverse events were not observed.

In a retrospective study based on autologous CIK cells, 40 patients with solid tumors were enrolled, including 10 patients with RCC after surgery and chemotherapy [20]. Percentages of CD3+, CD8+ and CD3+CD56+ lymphocyte subsets in patients’ peripheral blood were all significantly increased after CIK cell therapy. The 6-month OS rate was 70.0%, 1-year OS rate was 60.0% and 3-year was 57.5%. A favorable safety profile of CIK cells was observed in this study without severe adverse effect. Only two patients had fever and poor appetite and these symptoms could be relieved by simple treatments.

### 2.2. Clinical Studies on CIK Cells with Improvement of Culture Methods

Liu et al. reported a case applying modified autologous CIK (mCIK) cells for pleural metastasis of collecting duct carcinoma (CDC) [21]. CDC is a rare but highly aggressive RCC arising from the principal cells of the collecting duct. During the generation of mCIK cells, PBMCs were first primed with anti-CD3 antibody, and a mixture of IL-2, IL-18 and IFN-γ was subsequently added after 3 h. This generation method was distinct from the traditional way in which PBMCs are firstly cultured with IFN-γ, followed 24 h later by the addition of anti-CD3 antibody and IL-2. In every cycle, the patient received intrapleural infusion with modified CIK cells at a dose of 10 × 10^7^ once a day for 5 days. After three cycles of therapy, this patient showed relieved cough, dyspnea, chest distress, thoracalgia, and clearer and less pleural fluid. The only adverse reaction was fever after intrapleural infusion and the patient recovered 2 days later.

Another study investigated the efficacy of RetroNectin-activated autologous CIK cells (R-CIK) combined with conventional therapies (chemotherapy and radiotherapy) in patients with metastatic brain tumors [22]. RetroNectin is a 63 kD fragment of recombinant human fibronectin (also called CH-296) that promotes colocalization of virions and target cells to enhance the efficiency of retroviral mediated gene transduction [23]. RetroNectin-activated CIK cells were infused into the participants (1 patient with RCC, 14 patients with non-small cell lung cancer, 4 patients with breast cancer, and 1 patient with thyroid cancer). The OS rate in patients with all tumor entities was 21.4%. The disease control rate was 78.6%. The study also showed that the median PFS of patients receiving R-CIK was 7.7 months and the median OS was 12.6 months. The one patient with RCC achieved a CR without severe toxicities. OS of this patient was 14 months and PFS was 8 months. The results show RetroNectin could be a potential activator to augment the activity of CIK cells.

A study exclusively including patients with metastatic RCC was performed to explore whether the level of the underlying immune inhibitory factor myeloid-derived suppressor cells (MDSCs) was associated with the prognosis of patients receiving CIK cell therapy [24]. Autologous CIK cells were also activated with RetroNectin during generation. Twenty-nine patients received about 5 × 10^9^ CIK cells and 2 million IU IL-2 per day during the following 5 days in one cycle. The treatment schedule for the study consisted of at least 4 and at most 16 cycles in total. Of all evaluable patients, 4 exhibited a PR, 18 remained SD, and 7 showed PD, without CR observed. Moreover, the 1-year survival was 82.8% (24/29). Peripheral blood MDSCs were elevated in almost all RCC patients compared with healthy volunteers and decreased after CIK cell infusion. Also, patients with a relatively low proportion of MDSCs exhibited significantly prolonged survival, which indicated that MDSCs might serve as a potential marker for the prognosis of RCC patients receiving CIK cell treatment.

### 2.3. Clinical Studies on CIK Cells Combined with Dendritic Cell Vaccination

Dendritic cell (DC)-based vaccines represent a promising therapy that induces anti-tumor immune by initiating an antigen-specific T lymphocyte response [25]. DCs have high ability to present tumor antigens and compensate the lack of tumor antigen specificity of CIK cells. In a randomized controlled trial of 137 patients with postoperative localized and locally advanced RCC, 46 patients were treated with tumor lysate-pulsed DCs and autologous CIK cells, while 45 patients did not receive any postoperative adjuvant therapy [26]. After five intravenous infusions of tumor lysate-pulsed DC-CIK cells (3 × 10^8^ per infusion), an increase of the CD4+/CD8+ ratio and a decrease of CD4+CD25^high^ cells were observed. The metastasis and recurrence rates were significantly decreased, whereas the overall survival rates were significantly increased in the DC-CIK group. The achieved results showed that tumor lysate-pulsed DC-CIK cells could act as a promising postoperative adjuvant immunotherapy by preventing recurrence or metastasis.

In another study involving 121 patients with primary tumors (7 patients with RCC), patients received 1 × 10^7^ DCs for vaccination once a week for 6 weeks and 1 × 10^9^ autologous CIK cells six times within 4 days [27]. The delayed-type hypersensitivity skin tests detected a positive cell-mediated cytotoxicity response with the rate of 76.9%. Most patients achieved improvements in the quality of life (QoL). The treatment was safe and well tolerated and all side effects were mild and controllable.

Wang et al. reported a clinical trial evaluating the efficacy and safety of genetically modified DCs in combination with autologous CIK cells (gmDC-CIK) on patients with RCC [28]. Twenty-eight patients diagnosed with advanced clear cell RCC (ccRCC) (stageIIIB-IV) were enrolled in this study. On day 6 of cell preparation, DCs were pulsed with RNA encoding antigen muc-1 and survivin. In one cycle, patients received four subcutaneous injections of 2–5 × 10^7^ gmDCs on day 7, 9, 11, and 13 and intravenous infusion of 2–5 × 10^10^ CIK cells on day 11 and 13. The objective response rate (ORR) was 39% and a disease control rate (DCR) was 75%. DCR, but not ORR, was significantly related with cycles of treatment. Only one patient developed grade 1 fever and no other clinically significant side effect was observed.

In another clinical trial, 60 operable RCC patients were randomized to the autologous tumor lysate-pulsed DC-CIK (Ag-DC-CIK) cell treatment group or the control group, and 62 inoperable patients were randomly assigned to the CIK cell treatment group or control group [29]. In operable RCC patients, Ag-DC-CIK cell treatment significantly increased the 3-year disease free survival (DFS) and decreased the recurrence after surgery. Meanwhile, inoperable patients after CIK treatment achieved obviously longer 3-year OS and PFS than patients in the control group. The CD4+/CD8+ T cell ratio in peripheral blood increased after CIK cell treatment, and especially further increased in the autologous tumor lysate-pulsed DC-CIK treatment group. No grade 3 or 4 adverse reaction was observed during the cell transfusion.

Similarly, a study based on DC-CIK cell therapy recruited a total of 410 RCC patients after radical or partial nephrectomy [30]. One-hundred-and-fifty-four patients were included in the DC-CIK cell treatment group, while 256 patients were included in the IFN-α therapy control group. 2–5 × 10^6^ DCs per day were intradermally injected in the inguinal regions bilaterally and intravenous infusion of 3–6 × 10^9^ autologous CIK cells were performed once a day. A 6-day treatment was considered as one course, and the second course started after 1-month interval, for a total of three courses. The 3- and 5-year OS rates in the DC-CIK group (96% and 96%, respectively) were significantly more increased than that in the IFN-α group (83% and 74%, respectively). In addition, a statistically higher PFS was also observed in the DC-CIK group. The patients in the DC-CIK group exhibited good tolerance with just transient low-grade fever and fatigue.

A recent study compared the immune response induced by DC-CIK cell treatment and DC activated cytotoxic T cell (DC-ACT) treatment [31]. One-hundred-and-twelve patients with solid tumors (six with RCC) were assigned to the DC-CIK treatment group and 116 patients (six with RCC) were assigned to the DC-ACT group. In every infusion, patients received (1–3) × 10^7^ DC cells and (1–2) × 10^9^ autologous CIK cells. Each treatment consisted of two DC subcutaneous and three CIK intravenous infusions. Immune cells, such as CD3+ HLA-DR+ T cells and NK cells, and cytokines, like IL-2, IL-6 were significantly increased in DC-CIK group, whereas total CD3+ T cells, CD8+ T cells, CD3+ HLA-DR+ cells and IL-12 were remarkably increased in DC-ACT group. DC-ACT therapy decreased the ratio of IL-4/IFN-γ, IL-4/IL-12 and IL-6/IL-12 when compared with DC-CIK therapy. The results showed DC-ACT therapy promotes immune response towards Th1 cytokine profile, and DC-CIK and DC-ACT therapy may eradicate tumors through different pathways.

### 2.4. Clinical Studies on CIK Cells Combined with Targeted Agents

Yang et al. reported a case who was a 58-year-old male diagnosed with stage III clear cell RCC without metastasis [32]. After operation, this patient received combinatorial therapy consisting of autologous CIK cells and sorafenib because of the suspicious nodule identified in his left lung. The sorafenib dose was 400 mg twice a day and CIK cell infusion was conducted approximately every 2 weeks. However, adverse side effects, including grade 3 diarrhea, grade 3 fatigue and grade 2 hand-foot syndrome, were apparent and exceeded the patient’s tolerance. When sorafenib dose was decreased to 200 mg twice a day with CIK cell immunotherapy continued, the adverse effects became mild and well tolerated.

The nodule remained stable for approximately 6 months and then gradually decreased. Suspect metastases again emerged in the mediastinum and lung 6 months later, but the progress rate of these diseases was very slow. The patient maintained a high quality of life throughout the entire treatment process, with a KPS of 90 and greatly extended OS. These results suggest a potential combinatorial regimen consisting of sorafenib and CIK cells for metastatic RCC patients and indicate the safety of CIK cell-based immunotherapy.

Mai et al. retrospectively analyzed the efficacy of sunitinib/sorafenib in combination with autologous DC-CIK cells in 34 metastatic RCC patients after radical nephrectomy [33]. Fifteen patients in group 1 were treated with sunitinib/sorafenib monotherapy, while 19 patients in group 2 were treated with sunitinib/sorafenib in combination with DC-CIK cells. The median PFS and 3-year OS was significantly higher in group 2 and the cases of PD and deaths were less in group 2 than that in group 1. In addition, the 3-year OS was higher in sunitinib + DC-CIK group than that in sorafenib + DC-CIK group.

### 2.5. Clinical Studies on CIK Cells Combined with Immune Checkpoint Inhibitors

A phase I study aimed to assess the safety and clinical activity of the PD-1 blockade pembrolizumab in combination with autologous DC-CIK cells [34]. Thirty-seven patients with various solid tumor entities, including 8 with RCC, were enrolled in this study. Patients received more than eight cycles of intravenous infusion of pembrolizumab-activated autologous DC-CIK cells. The median OS and PFS were 270 and 162 days, respectively at the time of report. In RCC patients, objective responses (CR or PR) were observed in two of the eight patients, and the overall disease control rate in RCC patients was 75.0%. Treatment-related adverse reactions were observed in 20/31 patients. Two patients with bladder cancer and hepatocellular carcinoma, respectively, exhibited grade 3 or 4 toxicities, including fever and chills. All adverse reactions were reversible or controllable.

The anti-tumor activity of pembrolizumab-activated DC-CIK cells was then confirmed ex vivo. Autologous renal carcinoma cells were collected from one patient with PR and another with SD after treatment. Expression of PD-L1 was upregulated by DC-CIK cells and after being cocultured with pembrolizumab; the cytotoxicity and IFN-γ secretion level of DC-CIK cells were obviously increased.

Wang et al. reported an RCC case treated with the anti-PD-1 mAb pembrolizumab combined with CIK cell transfer [35]. This patient is an 80-year-old male with multiple metastases after partial nephrectomy. The tumor biopsy from this patient showed moderate CD3+ T cell infiltration, but no PD-1 or PD-L1 expression. After receiving four cycles of pembrolizumab combined with eight cycles of autologous CIK cell transfer, he achieved a CR. This patient developed gingivitis after the first cycle of pembrolizumab and pneumonia after the second cycle of pembrolizumab, for which he received systematic antibiotic treatment. No other severe adverse effects were observed. These findings showed the anti-tumor potency and safety of the PD-1 inhibitor in combination with CIK cell therapy on RCC.

The study design, clinical responses, adverse effects and conclusions of all clinical studies discussed above are outlined in Table 1.

## 3. Preclinical Researches to Improve the Anti-Tumoral Activity of CIK Cells

Ever-growing clinical trials have been conducted with CIK cells in cancer patients, owing to the relative easiness of CIK cell preparation ex vivo, low toxicity and GVHD activity, and their encouraging cytolytic activities against various tumor cells [15]. However, the clinical benefits of CIK cells reported in some studies are still limited and fall for inability of exerting a sustained and prolonged anti-tumor response. Researchers are exploring novel methods to modify CIK cell-based immunotherapies for a higher efficacy and more specific recognition against RCC. These preclinical researches conducted with CIK cells are concluded in Table 2.

Genetically modified CIK cells are of potential therapeutic value in the treatment of RCC. IL-7 is a 25kDa cytokine, which induced the proliferation of mature CD4+ and CD8+ T cells and also CIK cells [36]. Finke et al. used the adenovirus-enhanced CD3 receptor-mediated gene transfer for transfection with the IL-7 gene [37]. CIK cells transfected with IL-7 gene showed more IL-7 expression and a higher proliferation rate as compared with non-transfected cells. Expression of IL-7 also increased the production of tumor necrosis factor-α (TNF-α) by CIK cells and improved the cytotoxicity against RCC, malignant melanoma and colon carcinoma cell lines, indicating CIK cells transfected with an IL-7 gene expression construct may be valuable for adoptive immunotherapy on RCC. In another study, DCs were transduced with adenoviruses carrying human CD40L (Ad-hCD40L) to stimulate the anti-tumor activity of CIK cells [38]. CD40L is the ligand of CD40, a member of the tumor necrosis factor receptor family and is essential in antigen-presenting cells (APCs) activation and cytotoxic T lymphocytes (CTLs) generation [39]. DCs transduced with Ad-hCD40L had high expression of soluble CD40L and membrane-bound CD40L and showed a T-helper cell type 1 shift of expressed cytokines/chemokines. In addition, Ad-hCD40L transduction of DCs also significantly stimulated the proliferation of CIK cells and their cytotoxicity against RCC cell lines. This observation shows the immunomodulatory potential of genetically modified DCs for the CIK cell-based immunotherapy. 

**Table 2:** Summary of preclinical researches conducted with CIK cells on RCC.

Study	Method	Conclusion
Finke et al. [37]	CIK cells transfected with IL-7 gene	Improved proliferation rate and increased TNF-α production of transfected CIK cells; significantly higher cytotoxic activity against the RCC cell line
Hillebrand et al. [38]	DCs transduced with adenoviruses carrying human CD40L (Ad-hCD40L) + CIK cells	Co-culture of Ad-hCD40L DCs with CIK cells led to a significant stimulation of tumor-specific CIK cells, with increased proliferation and cytotoxicity
Sievers et al. [40]	Telomerase peptide pulsed DCs + CIK cells	Significantly increased cytotoxic activity against RCC cell lines and autologous, telomerase positive primary cell cultures
Zhang et al. [41]	An anti-TIGIT functional antibody + CIK cells	CIK cells with TIGIT blocked indicated increased proliferation, higher cytotoxicity against tumor cells expressing CD155 and higher expression of IFN-γ, IL-6, and TNF-α
Setiawan et al. [42]	Peptide P60+ CIK cells	CIK cells combined with P60 resulted in a significant decrease in the viability of renal and pancreatic cancer cell lines
Dehno et al. [43]	CIK cells treated with nivolumab and ipilimumab	CIK cells treated with nivolumab and ipilimumab had no remarkable effect on the viability of RCC cells; the combination of nivolumab and ipilimumab significantly increased the proliferation and IFN-γ secretion of CIK cells

IL-7: interleukin-7; TNF-α: tumor necrosis factor-α; DCs: dendritic cells; TIGIT: T cell Ig and ITIM domain; IFN-γ: interferon-γ; IL-6: interleukin-6.

Telomerase is a ribonucleoprotein enzyme that prevents the end of the chromosome from damage or fusion. Activation of telomerase is detected in nearly all human cancers. Telomerase peptide was added to pulse DCs and induce the tumor-specific lysis of CIK cells [40]. CIK cells had a significant increase in cytotoxic activity against RCC cells after being co-cultured with telomerase peptide pulsed DCs when compared with CIK cells without co-culture, that is 100% versus 41.7% at an effector-to-target (E/T) ratio of 60:1. The activated CIK cells also exerted specific cytotoxicity against autologous, telomerase positive primary cell cultures. Telomerase could, thus, serve as a specific tumor-associated antigen for RCC and allow the generation of antigen specific CIK cells.

T cell Ig and ITIM domain (TIGIT) is a newly identified inhibitory receptor expressed on T and natural killer (NK) cells [44,45]. A study showed that TIGIT was also expressed by CIK cells and interacted with CD155, the human poliovirus receptor expressed in many types of normal and cancer cells [41]. The anti-TIGIT functional antibody blocked TIGIT expression of CIK cells and promoted their proliferation. Cytotoxicity against CD155 positive tumor cells was enhanced with an increased level of cytokines, such as IFN-γ, IL-6, and TNF-α. However, inhibition of DNAX accessory molecule-1 (DNAM-1) reduced the elevated anti-tumor activity and IFN-γ level by TIGIT blockade. The results indicated that TIGIT shared the same ligand, CD155, as DNAM-1 and played an inhibitory role in CIK cell cytotoxicity. This finding provides a new molecular target for the improvement of CIK cell immunotherapy.

Peptide P60 has been shown to inhibit the immunosuppressive functions of regulatory T-cells (T-regs) by targeting towards the transcriptional regulator protein forkhead box P3 (FOXP3) [46]. As T-regs was shown to reduce the effectiveness of CIK cells against tumor cells, P60 was added to improve the cytolytic activity against renal and pancreatic cancer cells [12,42]. No increase in IFN-γ secretion and changes in the distribution of major effector cell populations in CIK cells was detected. However, P60 treatment resulted in a significant decrease in the viability of renal and pancreatic cancer cell lines co-cultured with CIK cells.

Recently, the anti-PD-1 mAb nivolumab and anti-CTLA-4 mAb ipilimumab were investigated if the two immune checkpoint inhibitors could stimulate the cytotoxicity of CIK cells against RCC cell lines [43]. Although nivolumab and ipilimumab combined with CIK cells had no remarkable effect on the viability of tumor cells, combination of the two antibodies significantly increased the proliferation and IFN-γ secretion of CIK cells. This finding indicated the possibility of combining immune checkpoint inhibitors with CIK cells as a potential treatment option for RCC.

## 4. Conclusions

The clinical studies based on CIK cells have shown big promise in the treatment of RCC patients, including those who are in the late stage or refractory to standard therapies. The MHC-unrestricted anti-tumor activity against a wide range of tumor types is one of the most important characteristics of CIK cells. CIK cells are also featured as their relatively low and easily controllable toxicities. In our previous report, the primary side effects of CIK cell therapy were grade 1–2 toxicities like fever, chills, fatigue, headache, and skin rash [47]. Low-grade fever ranged from 37.5 to 40 °C, was the most common adverse event and usually recovered without or with simple treatments [48]. In addition, CIK cells exhibited a reduced risk of GVHD, making CIK cells an ideal candidate for immunotherapy in both matched and mismatched recipients [49].

Many attempts have been made to optimize the treatment modality of CIK cell therapy on RCC. As the clinical studies described above, CIK cells combined with conventional therapies, DC vaccination, targeted drugs or immune checkpoint blockade could achieve better clinical benefits than the standard treatments alone. One of the most successful clinical studies that should be highlighted is reported by Zhao et al. [29]. In 122 patients with different stages of RCC, CIK cell treatment remarkably increased the 3-year survival no matter if it was in operable patients or in inoperable patients. This study suggested personalized CIK cell-based therapy would help to obtain clinical benefits in patients with different stages. Besides sunitinib and sorafenib, which show promising anti-tumor activity combined with CIK cells, other receptor tyrosine kinases, like c-Met, also play an important role in the growth of RCC. Increased expression of c-Met was observed in all RCC subtypes and associated with poor prognosis in RCC [50]. Balan et al. reported that c-Met inhibited immune cell-mediated killing of RCC cells by increasing the expression of PD-L1 [51]. Therefore, c-Met inhibitors may also exert synergistic anti-tumor effect with CIK cells. 

Moreover, a variety of preclinical researches have been developed and explored new target molecules and pathways to augment the efficacy of CIK cells’ anti-tumor activities. The newly identified inhibitory receptor TIGIT serves as a novel and encouraging target to increase the anti-tumor activity of CIK cells. With the elevated secretion of IFN-γ after TIGIT blockade, expression of PD-1 on CIK cells and PD-L1 on both CIK and tumor cells could also be detected to further develop the cytotoxicity profile of CIK cells by immune checkpoint blockade.

Although there is growing interest in CIK cells as an important cellular anti-tumor therapy on RCC, the heterogeneity in study design, clinical protocol and evaluation criteria of clinical studies all make it difficult to assess and compare the clinical efficacy. In 2010, the first international platform for the registration of clinical trials on CIK cells, IRCC, was established to standardize the clinical data collection [52]. The first update took place in 2015 and the second was published recently [47,53]. Further large-scale studies are required to evaluate the clinical efficacy of CIK cells and more efforts should be performed to identify the optimal CIK cell-based therapeutic combinations on RCC.

## Figures and Tables

**Table 1:** Clinical studies applying CIK cells for the treatment of RCC.

Study	Number of Patients	Therapeutic Approach	Clinical Response	Adverse Event	Conclusion
Liu et al. [21]	1 (1 RCC)	Modified auto-CIKs	Symptomatic improvement (relieved cough, dyspnea, chest distress and thoracalgia) and reduction of pleural fluid	Fever (~38 °C) but recovered 2 days later	The patient achieved partial success
Zhan et al. [26]	137 (137 RCC)	Group 1: tumor lysate-pulsed DCs + auto-CIKs Group 2: IFN-α Group 3: no adjuvant therapy	Increased CD4+/CD8+ ratio, decreased CD4+CD25^high^ cells and significantly higher 3- and 5-year OS rates	Controllable fever and fatigue in DC-CIK group	Postoperative immunotherapy with tumor lysate-pulsed DC-CIK cells may prevent recurrence/metastasis and increase OS rates
Zhang et al. [20]	40 (10 RCC)	Auto-CIKs	6-month, 1-year and 3-year OS rates were 70.0, 60.0 and 57.5%, respectively; median OS was 34.9 months; 6 recurrence, 12 metastasis and 15 deaths in all patients (2 recurrence, 5 metastasis and 3 deaths in RCC)	Controllable fever and poor appetite	CIK cell therapy can be an effective adjuvant instrument of the routine anti-tumor treatment
Cui et al. [27]	121 (7 RCC)	Tumor lysate-pulsed DCs + auto-CIKs	Improvements in the physical strength, appetite and sleeping status	Controllable fever, insomnia, anorexia, joint soreness and skin rashes	Tumor lysate-pulsed DC-CIK cells were safe without serious adverse side-effects
Zhang et al. [19]	20 (20 RCC)	Group 1: auto-CIKs Group 2: control	Significantly longer median PFS; 6 CR, 4 SD in CIK group and 5 CR, 3 SD, 2 PD in control group	Mild arthralgia, laryngeal edema, fatigue, and low-grade fever	CIK cell treatment could prolong survival in patients with RCC after radical nephrectomy
Wang et al. [28]	28 (28 RCC)	GmDCs + auto-CIKs	4 CR, 7 PR, 10 SD, 6 PD and 1 death	Flu-like symptoms with fever	DCR was significantly related with cycles of treatment
Wang et al. [24]	29 (29 RCC)	Auto-CIKs + IL-2	4 PR, 18 SD and 7 PD; 1-year survival was 82.8%; median PFS and OS were 7.7 months and 12.6 months, respectively	Grade 1 and 2 fever and grade 2 diarrhea	Auto-CIKs can induce regression of RCC; MDSCs can serve as a potential marker for the prognosis of patients receiving a CIK-based therapy
Li et al. [22]	20 (1 RCC)	RetroNectin-activated auto-CIKs + conventional therapies	1 CR, 5 PR, 9 SD and 5 PD; median PFS and OS were 7.7 months and 12.6 months, respectively (1 CR with an OS of 14 months and PFS of 8 months in RCC)	Fever	RetroNectin-activated CIKs combined with conventional therapies could improve the prognosis of metastatic brain tumor patients
Zheng et al. [30]	410 (410 RCC)	Group 1: tumor lysate-pulsed DCs + auto-CIKs Group 2: IFN-α	Significantly increased 3-, 5-year OS rates and PFS in DC-CIK group	Transient low-grade fever and fatigue	Adjuvant DC-CIK treatment after surgery prolonged PFS and reduced mortality
Zhao et al. [29]	122 (122 RCC)	In operable patients: Group 1: tumor lysate-pulsed DCs + auto-CIKs Group 2: control in inoperable patients: Group 1: auto-CIKs Group 2: control	Significantly higher 3-year DFS and decreased risk of post-operative disease progression and relapse in operable patients; Significantly higher 3-year OS rate, median OS and PFS in inoperable patients	Flu-like symptoms such as fever and fatigue	DC-CIK cells might be more efficient and personalized for the patients with tumor resection, and CIK cells could improve the prognosis for inoperable patients
Yang et al. [32]	1 (1 RCC)	Auto-CIKs + sorafenib	Metastasis remained stable	No serious adverse reactions	CIK cells combined with sorafenib could result in a synergistic effect
Mai et al. [33]	34 (34 RCC)	Group 1: sunitinib/sorafenib monotherapy Group 2: DCs + auto-CIKs + sunitinib/sorafenib	Significantly higher median PFS and 3-year OS rate; more SD and less PD and death	No serious adverse reactions in group 2; bone marrow suppression, oral ulcer, fatigue, and hand-foot syndrome	Sunitinib/sorafenib combined with CIK cells could significantly prolong the median PFS and 3-year OS
Wang et al. [35]	2 (1 RCC)	Auto-CIKs + pembrolizumab	1 CR (RCC) and 1 near-CR	No serious adverse reactions associated with CIK cells	Pembrolizumab in combination with CIK cells led to potent anti-tumor activity in RCC; CD3+ T cell infiltration in baseline tumor biopsies is a potential predictive biomarker
Chen et al. [34]	37 (8 RCC)	DCs + auto-CIKs + pembrolizumab	2 CR, 5 PR, 13 SD and 11 PD (1 CR, 1 PR, 4 SD, 2 PD in RCC); median OS and PFS were 270 and 162 days	All treatment-related adverse reactions were reversible or controllable; grade 3 or 4 toxicities, including fever and chills, were observed in two patients	Pembrolizumab-activated autologous DC-CIK cells were safe and effective in advanced solid tumors
Li et al. [31]	228 (12 RCC)	Group 1: tumor lysate-pulsed DCs + auto-CIKs Group 2: tumor lysate-pulsed DCs + cytotoxic T cells	Elevated percentage of CD3+ HLA-DR+ T cells, NK cells and cytokines such as IL-2, IL-6 in group 1; Elevated total CD3+ T cells, CD8+ T cells, CD3+ HLA-DR+ cells and IL-12 in group 2	--	DCs combined with cytotoxic T cells have more dominance to induce Th1 cytokine response instead of skewing toward the Th2 cytokine profile

Auto-CIKs: autologous CIK cells; DCs: dendritic cells; IFN-α: interferon-α; OS: overall survival; PFS: progression-free survival; CR: complete response; PR: partial response; SD: stable disease; PD: progressive disease; gmDCs: genetically modified dendritic cells; DFS: disease-free survival; DCR: disease control rate; IL-2: interleukin-2; MDSCs: myeloid-derived suppressor cells.
